# Death of Professor Randolph

**Published:** 1848-05

**Authors:** 


### DEATH OF PROFESSOR RANDOLPH.

The Philadelphia papers announce the death of Jacob Randolph, M. D., Clinical Professor of Surgery in the University of Pennsylvania, which took place in that city on the 29th of February. Dr. Randolph enjoyed a high reputation as a surgeon, and was universally esteemed by his brethren for the correctness of his professional deportment, and the uprightness of his entire life. As a lithritotist he was unrivaled in this country. A biographical memoir of the deceased is to be prepared by Dr. Norris, for the Philadelphia College of Physicians, of which Dr. R. was a member.

